# Monthly Summary

**Published:** 1896-03

**Authors:** 


### Monthly Summary.

SPITTING IN PUBLIC PLACES has become not only an intolerable nuisance, but a menance to health. It is well known that germs from dried sputum mixed with atmospheric air are easily respired, thus making consumption a communicable disease. The Board of Health of the city of New York, it is reported, will soon take action upon a report made by Dr. Herman M. Biggs, pathologist, and Dr. T. Mitchell Prudden, consulting pathologist, to the board. The report asseverates that it is time to put a stop to the filthy and disgusting practice of spitting on the floors of public buildings and conveyances. The report declares that it has long since been shown that the chief means for the transmission of consumption is the dried and pulverized sputum of persons suffering from this disease. Diphtheria, influenza and other diseases, the report says, are also easily communicated in this way during certain stages of the diseases. Catarrhal affections may be communicated through dried spittle mixed dust. These germs are likely to be gathered on the feet and on the skirts of women, the report says, and taken into private houses, where the most perfect ventilation will not stay their evil effect.

The report recommends the adoption of the following resolutions:

*Resolved*, That notices be posted in all public places and in all surface and elevated cars in this city, signed by the Board of Health, warning passengers against expectoration upon the floors of these conveyances, and further, that similar notices be posted in the stations of the elevated roads, warning against expectoration upon the platforms and stairs or on the floors of the stations.

*Resolved*, That similar notices be posted in the halls and assembly rooms of all municipal and federal buildings in the city.

*Resolved*, That the municipal authorities be requested to provide sufficient and proper receptacles for expectoration for such public places as are in their control, and that the managers of the elevated roads be requested to provide similar receptacles sufficient in number for their stations and platforms, and that in all cases these receptacles shall be kept in a cleanly condition.

*Resolved*, That the officers of the Manhattan elevated road be requested to give peremptory orders to their guards to refrain from,and to prevent so far as possible, expectorations from the trains into the streets, and to secure the enforcement of these orders.

In many cities, notices are posted in the street cars requesting passengers not to spit upon the floor, but until conductors are empowered with special police authority to enforce the regulation it is practically a dead letter. We think the time has arrived for the board of health to take such action as will prevent spitting in public places.—*Buffalo Medical Journal.*

---

INFILTRATION ANESTHESIA.—One of the great dangers of major operations is the administration of the anesthetic even when the anesthetists is skilled, and many a piece of surgery well done ends fatally through the evil effects of the sleep-producer. In minor surgery timid persons cannot stand even the momentary pain and some, too often of the so-called stronger sex, demand a total unconsciousness rather than submit to the momentary pain of an abscess opening or slight operation on the eye.

The use of cocaine has in a measure been of great assistance in these small operations, but of late Schleich of Berlin, in his article on "Schmerzlose Operationen," has devised a means of making the skin anesthetic for a short time in minor operations. Wurdemann, in reviewing this work in the

*Journal of the American Medical Association*, gives his experience with this new method in diseases of the eye, and Van Hook, in the *Medical News*, reports the result of his work in general surgery.

Schleich after some experimentation found that very small quantities of cocaine and morphia dissolved in a 0.2 per cent. salt solution and injected into and not under the skin, produced a localized anesthesia which lasted from ten to twenty minutes and often longer, allowing of cutting with no pain at all.

The skin is first made aseptic, then pinched up and the sterilized needle of the syringe containing the solution, which should be cold, is passed obliquely under the epidermis and a few drops injected until a white elevated wheal appears. The needle is withdrawn and inserted at the edge of this wheal and so on until an area as large as may be desired is made anesthetic. If the spot to be cut is first cooled by an ether or rhigolene spray, if it is the skin, and by a strong solution of carbolic acid, if it is the mucous membrane, the prick of the needle is not even felt, but in the case of the small needle which should be used, the pain is almost nil, especially when the skin is pinched. Wurdemann has used this method in operations about the eye and also in abscesses and felons with success. Schleich has even done celiotomies with it, but others might not dare to follow this lead.

The solution most usually employed is a grain and a half of cocain, one-third of a grain of morphia and three grains of sodium chloride in three ounces and three drachms of sterilized water. Two other solutions, one containing double the quantity of cocaine and the other one-tenth the amount, are also used.

The value of this operation is in the technique, and stress is laid on the point that the needle must not go beneath the skin. This process is capable of development and is worthy of a careful trial.—*Md, Med. Journvl.*

TO SECURE RUBBER DAM.—A year or so ago Dr. Hetrick gave a hint as to the value of sand-arach varnish to secure rubber dam to the teeth, instead of the painful silk ligature. It was one of the most valuable items I ever received, and if it has not been universally adopted it should be. It is absolutely demanded that dentistry should be made as easy as possible, and any neglect is scarcely less than criminal.

Many patients in a country practice are so situated that a prolonged course of treatment for aching or ulcerated teeth is out of the question. When operating for such a one, and an exposed living nerve is found, it can almost always be successfully removed at once by injecting a solution of cocaine, under strong pressure, so that it is forced up to the apex of the root, otherwise the removal will not be painless. When a fragment of these fresh pulps resist all efforts of the broach to effect removal, the heating of the Evans root-canal dryer and passing it up into the root brings the fragment away at once. The potassium and sodium compound of Dr. Schrier will also disintegrate one of these fresh pulps so it can be taken out at once.—Dr. Bergstresser in *Microcosm.*

---

CLEANING TEETH.—Many patients do not appreciate the cleaning of the teeth, because their dentists are in the habit of cleaning the teeth free, when there is no other work done. In thus donating this valuable service, the dentist receives no pecuniary return, and, indeed, very little thanks; for people are not disposed to value very highly what they get for nothing. On the subject of cleaning the teeth I think there is special need of public education People who appreciate the disastrous effects of caries, and who aim to have their teeth promptly filled on the appearance of cavities, are very often negligent about having them regularly cleaned, because they do not know the importance of this operation from a prophylactic standpoint. The patient is usually satisfied with a superficial cleaning of the anterior surface of the teeth.—J. W. O'Brien, in *Southern Dental Journal.*

TO RAISE THE STANDARD OF MEDICAL EDUCATION.—A meeting of the committee of the Association of the Ameriean Medical Colleges was recently held, and the following resolutions adopted, to present to the Association in June at San Francisco:

The committee agreed upon the following as necessary to admission to any of the seventy-one colleges forming the American Association:

English composition, in candidate's handwriting, not less than two hundred words, composition to include spelling, punctuation, paragraphing and construction. Examination in arithmetic, algebra through quadratics, and elementary physics. Latin to the extent of one year's study as indicated by Harkness's Latin Reader.

Graduates or matriculates of reputable colleges and high schools of the first grade, or normal schools established by State authority, or who have successfully passed the examination provided by the State of New York, may be exempt from the requirements named. Students deficient in one or more branches named as requirements shall have time until the beginning of the second year to make up such deficiency, provided, however, that students who fail in any two of the requirements in this second examination shall not be admitted to the second course.

A resolution was adopted to recommend to the Association that after the year 1895 no medical college shall be permitted to remain or become a member of the Association that does not provide either for a three-year course of eight months' study or a four-year course of not less than six months in each year.

A sub-committee was ordered to be appointed to prepare a curriculum of studies in the medical colleges providing for the minimum of time and lectures to be devoted to each one. This sub-committee will report to the general committee at the San Francisco convention.—"Southern Med. Record."

STERILIZATION OF DENTAL INSTRUMENTS.—The thorough and safe sterilization of dental and surgical instruments is a problem which is beset with no little difficulty. Most of our most potent antiseptic agents act detrimentally upon the steel surface, for which reason they must be excluded. High temperatures destroy or modify to a dangerous degree the delicate temper of many instruments, beside which the methods of heat sterilization require, in general, a special apparatus for the purpose, which adds complexity and difficulty to the process.

It has been shown by test and experience that the caustic alkalies, as well as their carbonates, possess marked germicidal properties. One of the most manageable and satisfactory agents of this class is liquor ammon. fort. Instruments soaked for a few minutes in a rather warm aqueous solution of ammonia are most beautifully cleansed thereby, without detriment to their polish or temper. We have no data bearing upon the relative efficacy of ammonia as a germicide, but believe it to be worthy of trial and scientific investigation in this relation.—*Cosmos.*

---

SALOL TOPICALLY.—Colombini ("British Medical Journal"). Dr. C. has been experimenting with regards to the local effects of salol dissolved in liquid vaselin. In the presence of alkaline fluids, or living tissues, salol appears to break up into salicylic and carbolic acids in the nascent state. When split up in this way, the author found that, while the good antiseptic action of each of the acids was manifested, their irritant action was absent. This, he thinks, is due either to the way in which they are set free, or to the small quantities of the acids evolved.

Clinically, it was found that salol in vaselin solution (the best solvent) did not irritate the skin or inflame ulcerated surfaces, which latter healed under the treatment without pain or local reaction.

The author therefore thinks there may be a useful field opened up in the local application of salol as a non-irritating and sufficiently powerful antiseptic.

A NEW SUBSTITUTE FOR GOLD.—A French technical paper, the *Journal det 'Horlogerie*, declares that a new amalgam has been discovered which is a wonderful substitute for gold. It consists of ninety-four parts of copper to six parts of antimony. The copper is melted and the antimony is then added. Once the two metals are sufficiently fused together, a little magnesium and carbonate of lime are added to increase the density of the material. The product can be drawn, wrought and soldered, just like gold, which it almost exactly resembles on being polished. Even when exposed to the action of ammoniacal salts or nitrous vapors it preserves its color. The cost of making it is about a shilling a pound avoirdupois.—*London News.*

---

IF we have confidence that we are a good dentist, we should surround ourselves with the evidence of our ability. A poverty-looking office is generally taken as the evidence of a poor dentist; untidiness as a slip-shod dentist; a dearth of literature as ignorance. We do not expect to find a clean dentist in an unclean office, an esthetic, approachable, genial fellow, in a dingy, musty, repulsive atmosphere; a pure, sympathetic, warm-hearted gentleman with a breath of tobacco smoke and stale beer. What we are is generally stamped on what we appear; in fact, what we appear to be is usually better than what we are, so that we have no excuse for a neglect that hides any good qualities we may possess.—*Items of Interest.*

---

SALT.—Under the Province of Gallicia, in Hungary, there exists a bed of pure rock salt 500 miles long, 20 miles wide and 250 feet in thickness. Consider what a sea once existed there, and how many ages of evaporation were required to deposit such a mass. A considerable portion of Western New York is underlaid by a great stratum of the same material. What effect has the abstraction of these immense quantities of salt had upon the condition of the sea water of to-day?—"Pract. and Advertiser."

COAL OIL IN THE LABORATORY.—There is a little wrinkle that I have made use of for years, and that is the substitution of coal oil for other oils in the laboratory. Used freely on the oil stone it keeps the latter fresh and clean, and there is left upon it no film of steel covering the surface of the stone and lessening its effectiveness. Used upon lathes, it removes all these gummy collections of old lubricants, and cleans journals and all other surfaces quite as well or better than lye. It will remove many of the discoloring accumulations from the surfaces of fine wood-work. Use it to soak old instruments in, to remove rust, etc. It has a dozen uses.—H. H. B. in *Dental Cosmos*.

---

CHANGES IN NERVE-CELLS—Lujano (*Lo Sperimento, University Med. Jour.*) concludes from experimentation that the activity of nerve-cells is accompanied by turgescence in the protoplasm of the cell substance. Fatigue produces a progressive diminution of the size of the cell. Ordinarily in moderate activity the nucleus does not change in volume, but in continued forced activity the changes induced are similar to those in the protoplasm, but not as marked. The first expression of cellular activity is thought to be an increase of chromatin, the late changes exhibit a diminution and diffuse distribution of it.

---

ARE RHUBARB LEAVES POISONOUS?—An inquest has just been held at Enfield, (London), upon a man, aged fifty-six, who had died after eating a pound and a half of rhubarb leaves boiled as a substitute for greens. A post-mortem was made, but it yielded no information.—*National Board of Health Magazine.*

---

PACIFIC COAST DENTAL CONGRESS.—The general committee of this body has determined that the next congress shall be held in San Francisco, in August, 1897, and that the American and Southern Dental Association shall be invited to meet at the same time and place, to hold separate or joint sessions as shall be deemed best.

THE WARM BATH IN INSOMNIA.—According to Dr. Eccles, the use of the warm bath for the purpose of producing sleep is very efficient if properly carried out. The bath should be administered in a room whose temperature is 65° to 70° F. The patient is made to stand with his head over the edge of the tub, and his head and face are then rapidly douched with water at 100° F. The cooling of the body by the air and the hot sponging of the head send the blood to the head, dilating the vessels of the entire brain. The entire body is then immersed, except, of course, the head, in a bath at 98° F., which is rapidly raised to a temperature of 105° to 110° In a few minutes the patient is taken from the bath, wrapped in warm blankets, and without exertion on his part, taken to his room. The blankets absorb the moisture; in his room the night clothes are put on, a warm bottle placed at his feet, and possibly some liquid food administered. The sedative and refreshing result is often most marked.—*The Sanitarian.*

---

Dr. Ferdinand Gorgas, Prof. of Principles of Dental Science, Surgery and Mechanism, University of Maryland, says: "My experience with Borine as a disinfectant, deodorizer and antiseptic has been very satisfactory both personally and in my practice."

Borine is termed the *Ideal Dental Antiseptic* possessing as it does in addition to its germicidal qualities those desirable ones of being *non-toxic and non-irritating.*

As a mouth wash it is without an equal. It hardens the gums, preserves the teeth and renders the breath pure and sweet. To wearers of artificial dentures it is an absolute necessity.

---

ACCIDENTS WITH NITRAT OF SILVER.—Table salt will neutralize nitrat of silver accidentally reaching the tissues or mucous membrane.—*Items of Interest.*

